# Chronic Anti-Coagulation Therapy Reduced Mortality In Patients With High Cardiovascular Risk Early In COVID-19 Pandemic

**DOI:** 10.21203/rs.3.rs-2252262/v1

**Published:** 2022-11-16

**Authors:** Mohamed S. Zaghloul, Momodou Jammeh, Andrew Gibson, Suhong Luo, Kelley Chadwick-Mansker, Qianjin Liu, Yan Yan, Mohamed A. Zayed

**Affiliations:** Washington University School of Medicine; Washington University School of Medicine; Veterans Affairs St. Louis Health Care System; Washington University School of Medicine; Washington University School of Medicine; Washington University School of Medicine; Washington University School of Medicine; Washington University School of Medicine

**Keywords:** COVID-19, Anti-Coagulation Therapy, Multi-Organ System Complications, In-Hospital Death, Intensive Care Unit Death

## Abstract

**Background:**

Coronavirus disease 2019 (COVID-19) is associated with provoked thrombo-inflammatory responses. Early in the COVID-19 pandemic this was thought to contribute to hypercoagulability and multi-organ system complications in infected patients. Limited studies have evaluated the impact of therapeutic anti-coagulation therapy (AC) in alleviate these risks in COVID-19 positive patients. Our study aimed to investigate whether long-term therapeutic AC can decrease the risk of multi-organ system complications (MOSC) including stroke, limb ischemia, gastrointestinal (GI) bleeding, in-hospital and intensive care unit death in COVID-19 positive patients during the early phase of the pandemic in the United States.

**Methods:**

A retrospective analysis was conducted of all COVID-19 positive United States Veterans between March 2020 and October 2020. Patients receiving continuous therapeutic AC for a least 30 days prior to or after their initial COVID-19 positive test were assigned to the AC group. Patients who did not receive AC were included in a control group. We analyzed the primary study outcome of MOSC between the AC and control groups using binary logistic regression analysis (Odd-Ratio; OR).

**Results:**

We identified 48,066 COVID-19 patients, of them 879 (1.8%) were receiving continuous therapeutic AC. The AC cohort had significantly worse comorbidities than the control group. On the adjusted binary logistic regression model, therapeutic AC significantly decreased in-hospital mortality rate (OR; 0.67, *p* = 0.04), despite a higher incidence of GI bleeding (OR; 4.00, *p* = 0.02). However, therapeutic AC did not significantly reduce other adverse events.

**Conclusion:**

AC therapy reduced in-hospital death early in the COVID-19 pandemic among patients who were hospitalized with the infection. However, it did not decrease the risk of MOSC. Additional trials are needed to determine the effectiveness of AC in preventing complications associated with ongoing emerging strains of the COVID-19 virus.

## Background

Early in the Coronavirus 19 (COVID-19) pandemic, this viral illness was a pervasive public health threat. In October 2021, the cumulative number of global cases had exceeded 240 million with nearly 5 million deaths. In addition to the interactions of the SARS-CoV-2 virus with human cells, severe COVID-19 disease was associated with a provoked hypercoagulable state that resulted in microvascular angiopathy and thromboembolic complications (1,2). Likewise, marked elevations in coagulation markers such as D-dimer were associated with severe COVID-19 disease states and increased mortality (3).

Cardiovascular risk factors such as hypertension, diabetes and coronary artery disease were highly prevalent among COVID-19 patients and carried a higher risk of morbid events as well as in-hospital mortality (4,5). Although acute respiratory distress syndrome was one of the hallmarks of the COVID-19 viral infection, multi-organ system complications (MOSC) was observed in patients with severe disease. A proportion of these patients presented with cardiovascular events, including myocardial infarction, arrhythmias, myocarditis and acute heart failure (6). Early in the pandemic, cardiac injury was a common occurrence and conferred a higher risk of in-hospital mortality (7). In addition to cardiac ischemia noted in autopsy studies, pathological micro-thrombosis was a common observation among patients with severe COVID-19 illness, suggesting a pro-thrombotic state may be contributing to these cardiovascular complications and overall poor recovery outcomes (8,9).

Since the early phase of the pandemic there has been an abundance of evidence to suggest that thrombosis alongside inflammation contributed to the poor outcomes observed with COVID-19 illness. The impact of anticoagulation in improving outcomes in already infected COVID-19 patients was therefore previously explored. In one multiplatform randomized controlled clinical trial, administration of therapeutic anticoagulation appeared to reduce thrombotic complications but not improve cardiopulmonary outcomes in already hospitalized critically-ill patients with COVID-19 (10–12). It is argued that hypercoagulable states associated with COVID-19 infection are often present during the early phase of the disease process, and this could in part account relatively modest benefits observed with therapeutic anticoagulation.

However, the data regarding the role of chronic anticoagulation in outcomes of COVID-19 has since remained conflicting. Parker et al. reported reduced rates of mechanical ventilation (8.5% vs 17.4%) in chronically anticoagulated patients with COVID-19 but a higher (40.2% vs 30%) mortality rate (13). On the other hand, Tremblay et al. demonstrated no mortality benefit in a propensity matched cohort of 241 patients receiving anticoagulation prior to COVID-19 infection (14). As such, we sought to evaluate the incidence of multisystem organ failure in individuals with high cardiovascular risks during the early phase of the COVID-19 pandemic. We hypothesize that patient who contracted COVID-19 during this time period, while already receiving therapeutic anticoagulation would have potentially benefited from reduced COVID-19-related complications.

## Methods

### Patient Population

We evaluated 48,066 COVID-19 positive U.S. Veterans who received care at the Department of Veteran Affairs (VA) during the early phase of the COVID 19 pandemic in the U.S. between March 1, 2020 and October 29, 2020. Patients were assigned to the AC group if they received a continuous prescription of therapeutically dosed anticoagulants at least 30 days prior to and 30 days after their initial COVID-19 positive test. The VA collects data from routine medical visits and compiles and stores them in a secure Corporate Data Warehouse (CDW). Patient information was de-identified before use in this study. This study was conducted retrospectively, and was approved with a waiver of informed consent by the institutional review board of the Department of Veterans Affairs St. Louis Health Care System.

### Study Variables

Patients with a COVID-19 positive test were identified from the VA COVID-19 Shared Data Resource, and confirmed from a combination of laboratory results and physician notes. Determination of therapeutic anticoagulant prescription dosing for assignment to the AC group was ascertained from the CDW outpatient pharmacy domain. Incidence of MSOC, between 5–30 days after initial COVID-19 positive status of stroke, limb ischemia, gastrointestinal (GI) bleeding, intensive care unit (ICU) death, ICU death, as well as composite MSOC outcomes were identified from International Statistical Classification of Diseases and Related Health Problems, Tenth Revision (ICD-10) diagnosis codes in CDW outpatient and inpatient encounters domains. Admission outcomes between 1–30 days after initial COVID-19 positive status related to hospitalization, ICU admission, death while in the hospital, death while in the ICU, hospital maximum length of stay, and ICU maximum length of stay were also extracted from the CDW inpatient encounters domain and the VA vital status database (date of death).

Study covariates included patient demographic information, including age, race, sex, and smoking status; prescription of medications including Aspirin, Insulin, and Statin; and clinical status, before and after initial COVID-19 positive test, of hyperlipidemia (HPL), chronic obstructive pulmonary disease (COPD), congestive heart failure (CHF), coronary artery disease (CAD), myocardial infarction (MI), diabetes (DM), hypertension (HTN), peripheral arterial disease (PAD), carotid artery stenting (CAS), chronic kidney disease (CKD), aortic valve replacement/ mitral valve repair (AVR/MVR), deep vein thrombosis (DVT), pulmonary embolism (PE), venous thromboembolism (VTE). Demographic information was collected from the CDW patient domain. The CDW outpatient pharmacy domain contained data for medication use. Patient clinical status was identified using ICD-10 diagnosis codes in CDW outpatient and inpatient encounter domains.

### Propensity matching

To reduce confounding caused by unbalanced covariates in anticoagulant group and control group, we also performed propensity score analyses. The patients in control group are about 53 times of the patients in anticoagulant group. Since there is little to gain in terms of statistical efficacy with over 4 controls to 1 case (15,16), we studied 1:1, 1:2, 1:3 and 1:4 matching. We used greedy nearest neighbor matching without replacement within 0.25 caliper method to form 1:1, 1:2, 1:3, 1:4 matching with propensity score, then chose the best matching ratio for entire cohort. Similarly, we performed the same propensity score analysis separately in hospitalized patients, as well as in ICU and non-ICU patients. Standardized mean difference pre- and post-1:1, 1:2, 1:3, 1:4 matching was performed to assess the quality of the propensity methods. From this, 1:2 and 1:4 matching appeared to be the best quality. We applied the same methods to hospitalized and ICU cohorts, and observed that 1:4 matching provided the best quality data for these cohorts. Therefore, to be consistence, we utilized 1:4 matching for all three cohort assessments, and then conducted conditional logistic regression analyses for all the study outcomes.

### Statistical Analysis

Characteristics of the overall study population, are reported as mean (95% confidence interval) or counts (percentage). T-tests for continuous covariates and chi-square tests for categorical covariates were conducted between the anticoagulant group and control group for each cohort. Multivariate binary logistic regression models, adjusted for covariates ascertained prior to the initial COVID-19 positive test, were used to compare various outcomes between those in the anticoagulation group and those in the control group. The unadjusted logistic regression model is summarized in eTable 1. Odds ratios with 95% confidence intervals are reported for those in the overall cohort and those in the hospitalized cohort. Effect modification for the composite MSOC outcomes was conducted for various covariates as well. All statistical tests were two sided, where a p < 0.05 or a 95% confidence interval that did not cross 1.0 was considered statistically. No imputation was conducted. All analyses were done using SAS Enterprise Guide, version 7.1 (SAS Institute Inc).

## Results

### Participants

**Figure 1:**
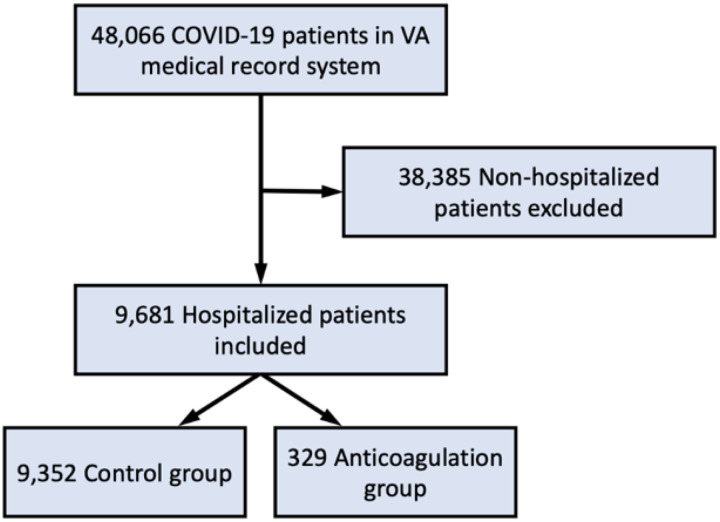
Study flow diagram. Out of 48,066 patients screened, we included 9,681 patients in our analysis. Among these patients, 9,325 were included in the control group and 329 were included in the anticoagulation group.

Of the 48,066 patients diagnosed with COVID-19, we identified 879 (1.8%) patients who received therapeutic AC prior to COVID-19 diagnosis. Among the subset of 9,681 (20.1%) patients who were hospitalized for COVID-19 in this population, the proportion receiving therapeutic AC was 329 (3.5%; Figure 1). Pharmacological agents used for therapeutic AC included apixaban (53%), rivaroxaban (24%), warfarin (14%), and dabigatran (9%).

Table 1 summarizes the baseline demographics including cardiovascular risk factors. The AC cohort had y more comorbidities than the control group. Most notably the AC cohort had 41.2% vs 16.0% chronic obstructive pulmonary disease (COPD), 43.9% vs 10% congestive heart failure (CHF), and 46.9% vs 15.3% coronary artery disease (CAD). The relative prevalence of comorbidities increased in the hospitalized versus non-hospitalized patients, including COPD (2.2-fold), CHF (3.2-fold), MI (3.3-fold), and CKD (3.3-fold). As expected, the prevalence of a prior history of thrombotic complications was higher in the AC cohort (DVT 18.0% vs 2.6%, PE 12.5% vs 1.3%), with relatively higher proportions noted in the patients who were hospitalized (DVT 2.9-fold and PE 3.3-fold).

### In-hospital outcomes

During the 30-day follow-up period, between the AC and control groups in patients who were hospitalized with COVID-19 (Table 2), there was an equivalent incidence of ICU admission (24.9% vs 23.6%, p = 0.74), as well as ICU length of stay (8.5 [4.5, 15.5] vs 9 [5.0, 15.5] days, p = 0.09). Likewise, between the two groups there was equivalent incidence in death during the hospitalization (10.6% vs 8.8%, p = 0.24), and death during the ICU stay (11.2% vs 10.2%, p = 0.55), despite the higher cardiovascular comorbidities in the AC cohort.

### Risk of adverse events in propensity-matched groups

Table 3 summarizes risk for adverse events in the propensity matched cohorts of the AC and control patients. There was a reduced risk of in-hospital mortality in patients who received AC (aOR 0.67, 95% CI [0.46 – 0.99], p = 0.04). However, no difference was observed in the incidence of death after ICU admission (aOR 0.93, 95% CI [0.64 – 1.37], p = 0.72), stroke (aOR 1.12, 95% CI [0.79 – 1.59], p = 0.52), and limb ischemia (aOR 1.09, 95% CI [0.30 – 3.91], p = 0.89). Interestingly, there was a higher incidence of GI bleeding in the AC cohort (aOR 4.0, 95% CI [1.16 – 13.82], p = 0.02).

## Discussion

Our study investigated whether continuous therapeutic AC decreased the risk of MOSC, ICU admission and in-hospital mortality in patients infected with COVID-19 during the early phase of the pandemic in the US. In the study cohort, the rate of in-hospital mortality was lower in the AC group compared to the control group, despite a higher incidence of GI bleeding. However, the rate of MOSC and ICU admission were similar between the two groups.

Several studies reported an increased risk of hypercoagulable state in COVID-19 positive patients (17–23). In a meta-analysis of 91 studies evaluating 35,017 COVID-19 patients, Mansory et al. found the overall incidence of VTE events in all hospitalized and ICU patients was 12.8% and 24.1% respectively (23). Reports showed hyper-thrombotic laboratory changes (e.g., d-dimer) in COVID-19 patients, and these changes are observed to be negative independent predictors of end-organ-damage and death (1,24–26). In addition, critically-ill COVID-19 patients manifest with a wide range of clinical symptoms that include interstitial pulmonary edema, ST elevation myocardial infarction, brainstem infarcts, gastro-intestinal mucosal bleeding, acute kidney failure, liver dysfunction, and skin petechial rashes (7,27–31). The mechanism by which COVID-19 viremia and cytokines induce thrombosis and inflammation is still not fully understood. However, some have postulated that hypercoagulability and MSOC in patient with COVID-19 results from a sequence of events that first starts with endothelial cell dysfunction and excess thrombin generation and fibrinolysis shutdown (32,33). Next hypoxia associated with severe COVID-19 can lead to further thrombosis via activation of hypoxia-inducible transcription factor-dependent signaling pathway (34). Lastly, immobilization during critically-ill states, can lead to blood flow stasis and higher incidence of in situ thrombosis.

Limited studies to date have assessed outcomes of therapeutic AC in patients with COVID-19, and the few that have provide contradictory findings. Early observational studies and a meta-analysis reported no difference in mortality in patients with COVID-19 who received AC therapy relative, but these studies suffered from small sample sizes, heterogenous patient populations, and unclear inclusion and exclusion criteria (14,35,36). In a recent meta-analysis of 11 studies investigating the impact of AC on mortality in a cohort of 20,748 hospitalized COVID-19 patients, AC therapy was found to be associated with a lower rate of in-hospital mortality in patients with COVID-19 (RR = 0.70, 95% CI 0.52 – 0.93, p = 0.01) (37). A large observational study including 4,389 patients from New York, demonstrated that therapeutic AC was associated with a 47% reduction in the risk of in-hospital mortality (aHR= 0.53; 95% CI: 0.45 – 0.62; p < 0.001) and 31% reduction in the intubation rate (aHR= 0.69; 95% CI: 0.51 – 0.94; p = 0.02) compared with no AC (38). In addition, Paranjpe et al. showed longer duration of AC treatment reduced the risk of in-hospital mortality (aHR of 0.86 per day; 95% CI: 0.82 – 0.89; p < 0.001) (39). On the other hand, AC was not shown to reduce the incidence or severity of MSOC such as acute kidney injury (13).

In the present study, patients who were on a continuous prescription of therapeutically-dosed AC therapy for at least 30 days prior to, and 30 days after their initial COVID-19 positive test, demonstrated reduced risk of in-hospital mortality when compared to control patients (aOR 0.67, 95% CI: 0.46 – 0.99, p = 0.04). Interestingly, our study observed that COVID-19 positive patients who received AC and had higher cardiovascular co-morbidities, actually had an equivalent incidence of MOSC. However, the potential benefits of AC in this higher risk population needs to be weighed against the risk of bleeding. Our study observed that AC therapy was associated with a higher incidence of GI bleeding, which is consistent with prior literature (25). As treatment protocols continue to evolve for patients afflicted with COVID-19, personalized assessments of the risks and benefits of AC therapy should be tailored to meet the most favorable anticipated outcomes.

The findings of this study should be interpreted with certain limitations. First, the associations explored were retrospective in nature and as such can include inherent biases associated with the study design, and method of data extraction and analysis. Second, there are factors that were not included in our propensity score matching that could also potentially impact mortality and morbidity. One such risk factor is smoking status, which was not discernable in the CDW. Third, within our study cohort we were unable to determine the primary cause of death for patients with COVID-19 during the early phase of the pandemic, which we found was often not clearly defined in the patient’s medical record.

## Conclusion

In summary, our study demonstrates that among U.S. veterans afflicted by COVID-19, chronic AC therapy reduced the risk of in-hospital death. However, AC therapy did not ameliorate the risk of MOSC. These findings suggest that MOSC in patients with COVID-19 are unlikely to be mainly determined by the patient’s pro-thrombotic state. Randomized control trials are needed to further evaluate the effectiveness of AC in preventing COVID-19-related MOSC, particularly in later and ongoing stages of this evolving viral illness.

## Figures and Tables

**Table 1.** Baseline characteristics of study population at the time of COVID-19 diagnosis

	Whole Cohort (n=48,066)			Hospitalized (n=9,681)		
	Control (n=47,187)	Anticoagulation (n=879)	P-value	Control (n=9,352)	Anticoagulation (n=329)	P-value
**Age**, yrs	59.0 (43.0, 71.0)	72.0 (67.0, 78.0)	<0.01	69.0 (59.0, 76.0)	73.0 (69.0, 80.0)	<0.05
**Race**						
white	31616 (67.0)	669 (76.1)	<0.01	5472 (58.5)	221 (67.2)	<0.05
black	14275 (30.3)	196 (22.3)		3646 (39.0)	100 (30.4)	
other	1296 (2.7)	14 (1.6)		234 (2.5)	8 (2.4)	
**Sex**						
male	38337 (81.2)	855 (97.3)	<0.01	8677 (92.8)	319 (97.0)	<0.05
**Smoking**						
current	7115 (15.1)	102 (11.6)	<0.01	1657 (17.7)	41 (12.5)	<0.05
former	10386 (22.0)	343 (39.0)		2738 (29.3)	126 (38.3)	
never	19163 (40.6)	400 (45.5)		4170 (44.6)	143 (43.5)	
**Comorbidities**						
HPL	25277 (53.6)	744 (84.6)	<0.01	6513 (69.6)	273 (83.0)	<0.05
COPD	7560 (16.0)	362 (41.2)	<0.01	2710 (29.0)	159 (48.3)	<0.05
CHF	4715 (10.0)	386 (43.9)	<0.01	2107 (22.5)	180 (54.7)	<0.05
CAD	7205 (15.3)	412 (46.9)	<0.01	2592 (27.7)	170 (51.7)	<0.05
MI	2374 (5.0)	145 (16.5)	<0.01	1067 (11.4)	80 (24.3)	<0.05
DM	14819 (31.4)	499 (56.8)	<0.01	4546 (48.6)	200 (60.8)	<0.05
HTN	25710 (54.5)	807 (91.8)	<0.01	7200 (77.0)	310 (94.2)	<0.05
PAD	1939 (4.1)	94 (10.7)	<0.01	804 (8.6)	42 (12.8)	<0.05
CAS	1520 (3.2)	94 (10.7)	<0.01	587 (6.3)	40 (12.2)	<0.05
CKD	5442 (12.1)	289 (32.9)	<0.01	2461 (26.3)	161 (48.9)	<0.05
AVR/MVR	2194 (4.6)	190 (21.6)	<0.01	839 (9.0)	91 (27.7)	<0.05
DVT	1212 (2.6)	158 (18.0)	<0.01	518 (5.5)	61 (18.5)	<0.05
PE	612 (1.3)	110 (12.5)	<0.01	270 (2.9)	54 (16.4)	<0.05
VTE	1577 (3.3)	222 (25.3)	<0.01	671 (7.2)	95 (28.9)	<0.05
**Medications**						
Aspirin	3641 (7.7)	116 (13.2)	<0.01	1222 (13.1)	60 (18.2)	<0.05
Insulin	2976 (6.3)	153 (17.4)	<0.01	986 (10.5)	66 (20.1)	<0.05
Statin	10689 (22.7)	536 (61.0)	<0.01	2974 (31.8)	194 (59.0)	<0.05

HPL, Hyperlipidemia; COPD, chronic obstructive pulmonary disease; CHF, congestive heart failure; CAD, coronary artery disease; MI, myocardial infarction; DM, Diabetes mellitus; HTN, hypertension; PAD, Peripheral arterial disease; CAS, Carotid artery stenting; CKD, Chronic kidney disease; AVR/MVR, aortic valve replacement/ mitral valve repair; DVT, Deep vein thrombosis; PE, pulmonary embolism; VTE, Venous thromboembolism. Data are presented as number (%) for categorical variables and mean (IQR) for continuous variables.

**Table 2.** In-hospital outcomes of ICU admission and death in COVID-19 patients

	Control	Anticoagulation	P-value
**Intensive Care Unit (ICU)**			
admission (%)	24.9	23.6	0.74
mean LOS (days)	8.5	9	0.09
**Death**			
during hospitalization (%)	10.6	8.8	0.24
during ICU admission (%)	11.2	10.2	0.55

**Table 3.** Adjusted multivariate logistic regression model of propensity matched analysis of COVID-19 associated MOSC between AC and Control groups

Outcome	OR	95% Confidence Interval	P-value
Stroke	1.12	0.79 – 1.59	0.52
Limb ischemia	1.09	0.30 – 3.91	0.89
GI bleeding	4.00	1.16 – 13.82	0.02
ICU death	0.93	0.64 – 1.37	0.72
In-hospital death	0.67	0.46 – 0.99	0.04

## Data Availability

De-identified datasets used and analysed during the current study are available from the corresponding author on reasonable request.

## Abbreviations

COVID-19: Coronavirus disease 2019
AC: Anti-Coagulation Therapy
MOSC: Multi-Organ System Complications
VA: Veteran Affairs
CDW: Corporate Data Warehouse
ICU: Intensive Care Unit
ICD-10: International Statistical Classification of Diseases and Related Health Problems, Tenth Revision
GI: Gastrointestinal
HPL: Hyperlipidemia
COPD: Chronic Obstructive Pulmonary Disease
CHF: Congestive Heart Failure
CAD: Coronary Artery Disease
MI: Myocardial Infarction
DM: diabetes mellitus
HTN: hypertension
PAD: Peripheral Arterial Disease
CAS: Carotid Artery Stenosis
CKD: Chronic Kidney Disease
AVR/MVR: Aortic Valve Replacement / Mitral Valve Repair
DVT: Deep Vein Thrombosis
PE: Pulmonary Embolism
VTE: Venous Thromboembolism
